# Stock market reaction to product-harm crisis response strategies

**DOI:** 10.1371/journal.pone.0290548

**Published:** 2023-08-24

**Authors:** Sujuan Zheng, Guangqing Yang, Shuhan Chen

**Affiliations:** 1 School of Finance, Fujian Jiangxia University, Fuzhou, China; 2 School of Economics and Management, Minjiang University, Fuzhou, China; 3 School of Economics & Management, Fuzhou University, Fuzhou, China; Swinburne University of Technology - Sarawak Campus, MALAYSIA

## Abstract

Product-harm crises have detrimental effects on firm’s sales, reputation, and financial value, requiring crisis managers to promptly adopt appropriate response strategies to mitigate these impacts. Situational Crisis Communication Theory (SCCT) guides managers to align responsibility attribution with response strategies. Using Chinese listed firms’ product-harm crises sample from 2015 to 2021, this study analyzes the stock market’s reaction to different response strategies. The event study method reveals that a passive strategy is more effective during the disclosure stage, and accept+no recall and deny+recall are conforming strategies during the initial response stage. Additionally, firms with a crisis history should assume greater responsibility when developing response strategies for product-harm crises, as crisis history amplifies negative effects. The results provide recommendations to help managers formulate appropriate strategies.

## Introduction

A Product-harm crisis is an incidental and widely publicized event about a product that is defective or dangerous to consumers [1]. This definition has been widely recognized by scholars. Dawar and Pillutla (2000) [2] provide a more comprehensive definition of a product-harm crisis, characterizing it as a multifaceted situation where a product exhibits defects, fails to meet safety standards, or poses potential dangers. With product diversification and more stringent regulatory standards, product-harm crises have become a common occurrence, for example, Samsung cell phone battery explosion in 2016, Chinese milk powder melamine in 2016, VW tailpipe emission scandal in 2015, U.S. Johnson & Johnson talcum powder cancer fiasco in 2018, etc. Such crises are among the most prevalent threats faced by organizations, which have significant consequences, including a drop in sales, a decrease in share value, loss of reputation, decline in public trust, and the potential to impact the entire industry [3–7]. When a product-harm crisis occurs, crisis managers eager to implement appropriate response strategies to alleviate its negative impact.

Scholars have proposed several strategies on how to deal with product-harm crises. Siomkos and Kurzbard (1994) [1] proposed four product-harm crisis response strategies, including denial, involuntary product recall, voluntary product recall and super effort. Super effort strategy means that firms maintain a high degree of candor in crisis communications to maximize consumer protection. Coombs (2007) [8] proposed Situational Crisis Communication Theory (SCCT) as a guideline for post-crisis communication to help managers effectively protect the firm’s reputation. SCCT comprises three key elements: crisis situation, crisis response strategies, and the matching system of crisis situation and crisis response strategies. It demonstrated that response strategies included four crowds of deny strategies (consist of attack the accuser, denial and scapegoat), diminish strategies (comprise of excuse and justification), rebuild strategies (comprise of compensation and apology), and lstering strategies (reminder, ingratiation, victimage), as well as emphasized the significance of aligning responsibility attribution with the assumed responsibility in crisis response [8]. According to SCCT, stakeholders’ responsibility attribution of a crisis is influenced by the crisis type and crisis history. Crisis types consist of victim crisis, accidental crisis, and intentional crisis. Different types of crises can be categorized based on their causes and the level of responsibility attributed to the firms involved. Victim crises, including natural disasters, false rumors, workplace bullying, and product tampering, often holding firms to minimal primary responsibility. Accidental crises arise from challenges, safety accidents, and product failures due to technical issues, leading to relatively minor level of responsibility for firms. Intentional crises, involving financial fraud, accidents, and product crises caused by human factors and organizational misconduct, impose significant responsibility on the firms involved. As defined in the SCCT, most product-harm crises are intentional crises and a few may be accidental crises.

What are the appropriate crisis response strategies that align with responsibility attribution when facing a product-harm crisis? Based on the SCCT theory, this paper tries to investigate and offer effective crisis management strategies, with a specific focus on their impact on the stock market. Extensive prior literature has demonstrated that organizational misconduct and corporate irresponsibility behaviors led to evident negative outcomes [9–11] and triggered adverse stock market reactions [12–15]. Product-harm crises, as a form of organizational misconduct, commonly result in are organizational misconduct and bring negative stock price reaction. Given that maintaining stock price stability is one of the key tasks for managers, it becomes essential to provide them with well-informed suggestions on crisis response strategies from the perspective of stock market reactions.

Numerous studies have delved into product-harm crises, concentrating on areas such as financial markets, marketing, and brand equity. From a consumer perspective, scholars agreed that product-harm crises had a negative impact on corporate brand equity [2, 6, 16, 17]. From an investor perspective, scholars identified the negative impact of product-harm crises on the stock market [18–23]. From marketing perspective, scholars suggested that product-harm crises adversely affected marketing effectiveness [6, 24] and could have spillover effect on other competitors in the same industry [25, 26].

Although there has been rich literature on the stock market reaction to product-harm crises, most of them focused on product recall, including the timing of product recalls, remedy measures and marketing strategies. Less research has been conducted on the stock market reaction to firms’ attitudes toward taking responsibility at the onset of a product-harm crisis. In this paper, we divided firm’s responses into two stages: disclosure stage and initial response stage. At disclosure stage, we find that passive strategy is more effective than proactive strategy. A proactive strategy entails a company voluntarily disclosing its crisis information prior to its recognition by the media or other stakeholders. Conversely, the passive strategy involves the situation where the crisis information is initially revealed by external sources such as the media or stakeholders, rather than being initiated by the company itself [16]. At initial response stage, following the approach suggested by [27], we design a 2(deny vs accept) *2(recall vs no recall) factorial model to distinguish the firm’s attitude towards the product-harm crisis and find out matched response strategies. In addition, we further examine the impact of crisis history on the effectiveness of crisis response strategies and empirically support SCCT theory that firms with crisis history should assume a higher level of responsibility.

The remainder of this article is organized as follows. In section 2, we present overviews of previous literature on financial impact of product-harm crises and crisis response strategies, and develop the hypotheses. Then, we describe research methodology and data analysis in section 3. Section 4 shows the empirical results. Conclusion and discussion are presented in Section 5.

## Literature review and hypotheses

### Theoretical analysis model

**Fig 1:**
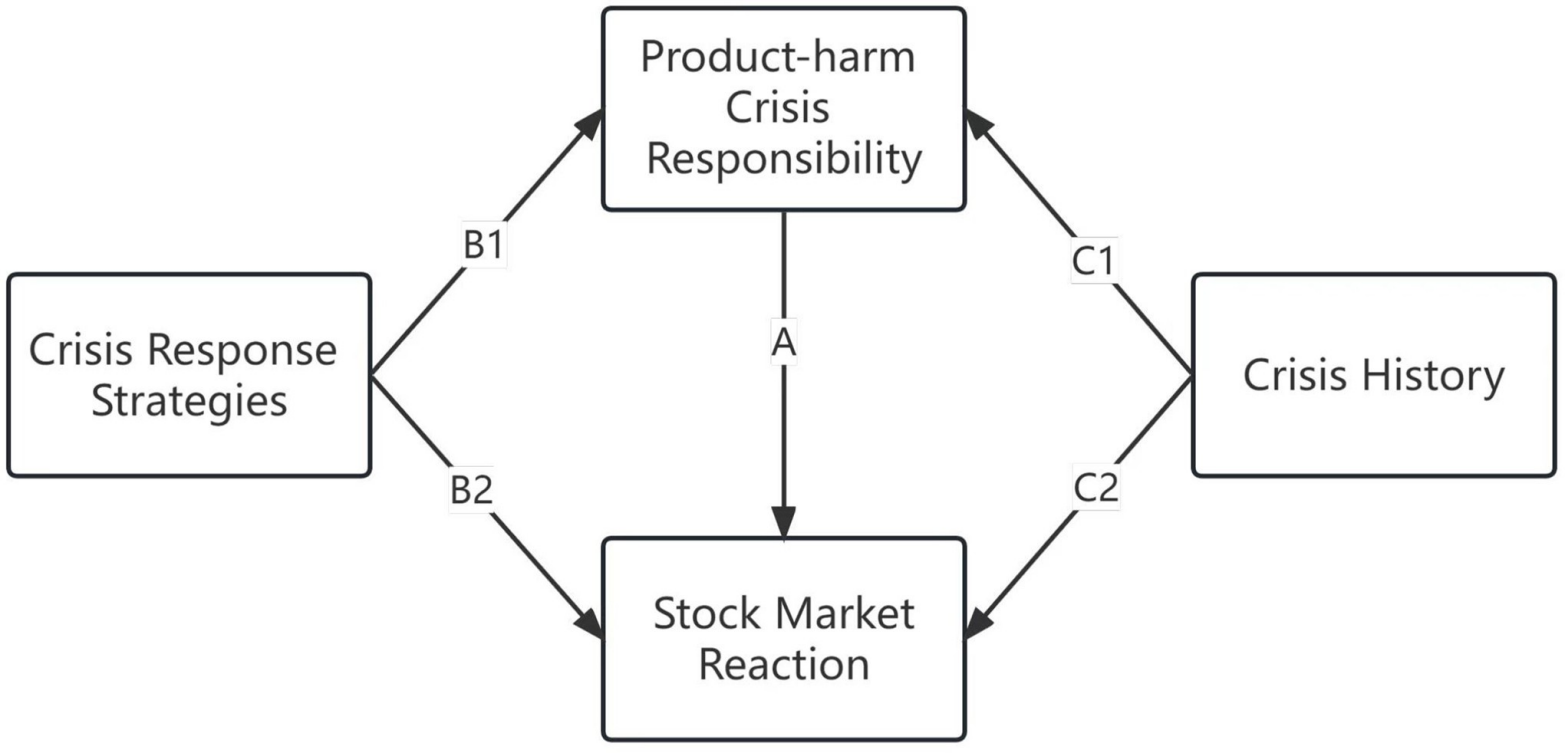
Relationships in product-harm crisis analysis model.

Based on the SCCT theory, this study illustrated the relationships between the key variables in a product-harm crisis and proposes research hypotheses. The discussion of the research hypotheses is mainly oriented to Fig 1. Fig 1 refers to Coombs’ crisis situation model [8] to present the relationships between the variables of product-harm crisis responsibility, crisis response strategies, crisis history, and stock market reaction.

Product-harm crisis responsibility implies stakeholders’ responsibility attribution for a crisis. Crisis response strategies involve what managers say and do after a crisis to reduce negative impact, including proactive strategy and passive strategy at disclosure stage, strategies combination of accept/deny and recall/no recall at initial response stage. Crisis history mentions the firm has similar crisis event before. Stock market reaction entails abnormal changes in stock returns at the onset of a product-harm crisis. Arrow A indicates the stock market reaction to product-harm crises responsibility. Arrow B1 represents that crisis response strategies build the attribution of responsibility for product-harm crisis. Arrow B2 indicates that crisis response strategies reduce the negative impact of product-harm crises. Arrow C1 shows the effect of crisis history on attribution of responsibility for product-harm crises. Arrow C2 shows the direct and indirect effects of crisis history on stock market reaction.

### Financial impact of a product harm crisis

Product-harm crisis is one type of negative publicity indicates the firm’s product is defective that fail to meet the industry standards, regulatory requirements, consumers’ need, or even cause the life, health and safety problems of consumers [1, 2]. As its definition shows, product-harm crisis will cause physical threat and psychological threat to consumers directly [8]. Both physical threat (for example, eating contaminated food, taking substandard drugs) and psychological threat (feelings of being cheated and disrespected) make consumer reputation shift from favorable to unfavorable which will damage firm’s image and reduce product sales in future [8]. In addition, product-harm crises can also pose financial threat and reputation threat to firms from investors’s perspective [20, 27, 28]. On the financial side, a product-harm crisis bring about a decline in sales, negatively impacting firm revenues and is often accompanied by product recalls, compensation, and regulatory punishment, all of which can result in economic losses for the firm and decrease investors’ interest [27]. On the reputation side, investors often associate a product-harm crisis with poor business operations, such as supply chain or operations-related deficiencies [29], all of which leads to a loss of investor confidence [20].

Product-harm crises give the public reasons to related the firms with bad behavior and threat the firm’s reputation equity. As the theory of efficient market emphasizes, firm’s stock price will react to the crises information promptly [30] and take a fall. Scholars have rich research on market reaction to product-harm crisis in automobile industry, the Consumer Packaged Goods industry, and the pharmaceutical industry [6, 20, 21, 24, 31, 32]. They have demonstrated the negative impact of a product-harm crisis on the firm’s stock prices when crisis firm make product recall announcement [20, 31, 33]. Product recalls generate threat on firm’s reputation and result in significantly negative abnormal returns for firms [22]. Product-harm crises are among a firm’s worst nightmares which can cause losses of major revenue and market-shares [26].

Although it is difficult to examine the long-term loss of product-harm crisis on the firm’s brand and reputation, the short-term impact on the firm’s financial value can be demonstrated through the event study method, using the daily stock prices [21]. Event study method is powerfully designed to evaluate the impact of an emergency on the firm’s market value and support to infer whether there is abnormal stock price effect related to unexpected events, usually to examines the stock price reaction to disclosure or announcement of emergencies. We propose:

*H*_*1*_: *A product-harm crisis will bring a negative abnormal return on stock price*.

### Proactive strategy vs passive strategy

Given the adverse influence of product-harm crisis on the firm’s reputation and financial performance on the stock market, how to respond appropriately at the onset of a product-harm crisis is absolutely a critical problem for the managers to deal with. The time they take to deal with product-harm crises depends on several factors [34]. Crisis timing strategy theorized the time of the crisis organization disclosure information determines the effect of crisis management [16]. It defines proactive exposure as an organization disclosing a crisis event before the media and other subjects. Using proactive strategy guaranteed the organization to explain the objective information about the specific event simply and naturally [35]. Alternatively, passive strategy means that the crisis event is first disclosed by the media or other subjects. In this case, taking a crisis response strategy confirming to the crisis attribution will be rational.

Researches on the effect of proactive strategy and passive strategy are abundant and divergent [20, 36]. Viewpoints can be concluded from two perspectives: the consumer perspective and the investor perspective. From the consumer perspective, previous research has indicated that a proactive strategy is more effective than passive strategies. This is because a proactive strategy conveys a more positive signal to consumers, which can lead to more favorable outcomes [2, 8, 36]. On the one hand, consumers perceived the firms taking proactive strategies as being of reliable, honest characteristics and recognized the firm cared about their safety and health. This kind of trust in the firm and perception of being concerned will help to increase the consumers’ confidence when they make purchase decision in the future [2]. On the other hand, proactive strategy conveyed that crisis firm was responsible for their behaviors and concerned about the consumers, which was a definitely good response to alleviate the negative mood at the onset of a product-harm crisis when the consumers are intuitive and emotional [8]. However, the outcome of empirical studies from the investor perspective shows contrary conclusions. They demonstrated proactive strategies will induce more loss in the stock market than passive strategies [20]. Firstly, investor pay attention to the firm’s healthy cash flow in the short term [20]. Proactive strategy signaled the firms will make product recall or be punished by the regulatory authorities, both of which indicated the financial loss directly. Secondly, investors may interpret proactive strategy as an extremely sever product-harm crisis that the firm can not control but only to choose to act hastily to diminish the hidden financial loss.

In this article, we focused on studying stock market reaction to crisis response strategies from investor’s perspective. Proactive strategy may convey poor information to investors and suggests that the product-harm crisis is severe and out of control, leading investors to believe that managers can do nothing but declare the event to the public, issue product recalls, or face punishment by regulatory authorities according to securities law [20, 26]. Otherwise, managers will first consider taking passive strategies to alleviate the negative impact on the firm’s reputation and market value, instead of adopting proactive strategy [2, 37]. Thus, most product-harm crises were disclosed passively, mainly published by consumers’ complaint, regulatory authorities’ findings or investors’ interactive questions. So we proposed:

*H*_*2*_: *At the onset of a product-harm crisis*, *proactive strategy brings a more negative impact on crisis firm’s market value on the stock market than passive strategy*.

### Matched response strategies

At the onset of a crisis event, stakeholders’ first reaction is to search for the cause and then make crisis responsibility attributions immediately [8]. In the process of seeking attributions, stakeholders will form their own views and emotions on crisis events. When stakeholders perceive the crisis as a preventable event, such as human-error product harm or organizational misdeeds, anger emotions are triggered, more responsibility is likely to be attributed to the firm and result in greater reputation loss; when stakeholders recognize the crisis as accidental or innocent, sympathy emotions dominate their behaviors, and crisis responsibility attributions may be minimal or weak [8, 38].

Most product-harm crises are generally considered to be man-made and intentional. Absolutely, stakeholders’ anger emotion will be triggered and evoked strong attributions of crisis responsibilities that conduce high reputation loss. All such negative impact will be reflected on the stock market, shown as the decline of the stock price [20, 24, 27, 39]. The managers required to take appropriate crisis response strategies to communicate with the stakeholders effectively, in order to express their responsibility and reduce the damage. The common practices mainly include making announcement, providing official declaration or issuing a statement on the official microblog.

Primary response strategies are classified as deny strategies, diminish strategies, and rebuild strategies [8]. Stakeholders often clarify a firm’s crisis response strategies by interpreting the text content and expression intonation used in their communications [40]. Based on the response strategies of a product-harm crisis, the text content and intonation can be classified into two dimensions: denial/acceptance and recall/no recall [8]. Stakeholders will use these dimensions to judge the company’s attitude toward crisis responsibilities. Acceptance strategy implies the crisis firm directly admit the truth of the product-harm crisis and is willing to take more responsibility [8]. Deny strategy indicated the firm announced that crisis did not occur or product inspection standard was not completely objective, or the event is not dangerous, or the relevant product has been stopped to produce [8]. As a whole, deny strategy suggest the firm decide to take less responsibility for a product-harm crisis. Recall strategy refers the firm explicitly propose product recall in the announcement, which conveyed taking more responsibility for the crisis [20]. Otherwise, crisis firms who have never mentioned the word of product recall or any recall measures signified there is no recall and represented the firm’s less responsibility for a product-harm crisis.

SSCT pointed that more attributions of a crisis should be matched with crisis response strategies with more responsibilities [8]. However, this does not demonstrate that the more responsibilities the company takes, the better effectiveness of crisis response strategies will be. Instead, effective crisis response strategy should confirm with the stakeholders’ crisis attributions appropriately [38, 41]. Both underconfirm (taking less responsibility than stakeholders’ expectation) and overconfirm (taking more responsibility than stakeholders’ expectation) are inefficient and will bring higher loss in firm’s reputation and market value. Underconfirming response strategies shows the firm’s lack of sincerity in taking responsibility which will reduce stakeholders’ trust in the firm, while overconfirming response strategies lead to the stakeholders’ suspicion of there are some other unknown information about the crisis or the crisis is more sever than it appears. Just as Bundy and Pfarrer proposed the more an organization’s response strategy matches stakeholders’ situational attributions of crisis responsibility, the lower loss caused by the crisis [38]. We proposed:

*H*_*3*_: *comparing with mismatched response strategies*, *matched response strategies are more efficient in reducing the negative impact on the firm’s value at the onset of a product-harm crisis*.

Assessing crisis attributions correctly is the prerequisite before making an effective response strategy when a product-harm crisis happened. In order to assess crisis attributions effectively, managers demanded to consider firm’s previous performance fully [42], especially crisis history.

Crisis history is a sign of consistency [8]. If a firm has a crisis history, stakeholders may believe that there is some underlying problem in the firm that has led to the recurrence of the crisis. Crisis history can also lead stakeholders to associate the firm with "repeat offenders" [43]. Therefore, crisis history makes stakeholders attribute more responsibility of crisis to the firm itself.

In addition, crisis history is often associated with corporate control [43], which means that the current crisis is not a isolated incident, but rather part of a pattern of behavior within the firm itself. As scholars have proposed, crisis history is a source of halo effect [44], and Velcro-like effect, causing additional reputational damage [45]. Its negative impact on firm reputation can accrue over time [46]. Thus, crisis history created more attributions of responsibility [47]. SCCT recognized that when crisis history existed, crises are classified into clusters with higher attributions of responsibility [8].

Crisis history is an intensifier of crisis attribution at the onset of a product-harm crisis [8]. Product-harm crisis firms with crisis history are more likely to be associated with intentional human errors and trigger external dissatisfaction [8]. Firm with product-harm crisis history should take more responsibilities to deal with a new product-harm crisis when making response strategy, comparing with the firm without crisis history. Thus, we proposed:

*H*_*4*_: *Firms with crisis history have better take more responsibilities when making response strategy at the onset of a product-harm crisis*, *comparing with the firms without crisis history*.

## Data and methodology

### Data

Our sample covered all listed firms in the Shanghai and Shenzhen Stock Exchanges, reported with product-harm crisis news from 2015 to 2021. We filter these firms through the news information table of listed firms in the China Stock Market and Accounting Research (CSMAR) database, and then collect supplementary details of the beginning and development process of each specific product-harm crisis event through Baidu search engine and CNINFO (http://www.cninfo.com), as Baidu search engine covers every news reported in China, and CNINFO is a listed company information disclosure website designated by China Securities Regulatory Commission.

Firstly, we entered keywords including "product recall", "unqualified product", "product safety", "quality problem", "exceeding the standard", and "food safety" in the news information table, and obtained all news information containing the above keywords.

Secondly, we excluded news information which was obviously irrelevant to the product-harm crisis, such as positive information about the firms taking measures to improve product safety. We also disregarded observations if such had a special treatment status (known as “ST stock” or *ST) when the product-harm crisis new was reported. Additionally, observations of firms in the finance industry were excluded from our sample due to their high sensitivity to government regulations.

Thirdly, as the qualified samples were identified, we read the news item by item to sort out the details of each specific product crisis, including the earliest reported by media and the relevant listed firms’ responses (such as company statements, micro-blog replies, announcements, etc.) to the event, combing with supplementary information from Baidu search engine, CNINFO, micro-blog, company official website, regulator official website.

Finally, we obtained 232 samples of product-harm crisis released in the China Stock Market and Accounting Research (CSMAR) database from 2015 to 2021. In order to get the ideal setting for an event study, we conducted secondary screening on the sample and eliminated sample events with confounding effects, who had other events during 10 trading days before and after the disclosure date of product-harm crisis, such as unexpected dividend or income announcement, acquisition bidding, merger negotiation, change of key management personnel, reorganization, joint venture, major contract winning, major labor dispute, major liability litigation and major new product release, etc. Because those other events could cause abnormal fluctuation of stock price, we cannot identify the influence induced by the product-harm crisis. In addition, we eliminated the samples with ambiguous time, insufficient estimation window and repeated suspension and resumption of trading. At last, our sample consists of 169 product-harm crises reported in the China Stock Market and Accounting Research (CSMAR) database from 2015 to 2021.

### Sample descriptions

Product-harm crisis events are revealed by several channels, most including the regulatory authorities, the firm itself, and the media. In China, product safety is administered by two major government agencies of National Medical Products Administration and State Administration for Market Regulation. The National Medical Products Administration is responsible for the safe issue of drug, medical device and cosmetics. The State Administration for Market Regulation is responsible for product safety and industrial product, involving foods, automobile and related equipment, housewear & furnishings, household appliances. Table 1 shows representative product-harm crises in different industries in China, from 2015 to 2021.

**Table 1:** Representative product-harm crises in China.

Industry	company	year	crisis description
**Pharmaceuticals**	Yunnan Baiyao Group Co., Ltd.	2015	National Medical Products Administration found that the unqualified ginkgo biloba extract purchased by Yunnan Baiyao was used to produce ginkgo biloba preparations.
	Chang Chun High and New Technology Industries (Group) Inc.	2021	Xinhua Viewpoint reported that the so-called "heightening needle" popular in the market is actually growth hormone. The next day after the article was released, the stock price of Changchun Hi Tech, which is mainly engaged in "heightening needle", went down the limit.
**Food**	JinJian Cereals Industry Co., Ltd.	2016	Sohu News released "62 students in a school in Huaihua are suspected of being sent to the hospital for vomiting due to drinking milk", which disclosed that many students in Huaihua were sent to the hospital for acute gastroenteritis diagnosed by drinking "Jin Jian" milk.
	Lanzhou Zhuangyuan Pasture Co., Ltd.	2018	The State Administration of Market Supervision and Administration issued that the samples of Zhuangyuan Pasture’s wholly-owned subsidiaries were unqualified, and required to find out the product flow direction and recall
**Automobile**	Zhongtong Bus Holding Co., Ltd	2017	Zhongtong Bus Holding Co., Ltd. filed a recall plan with the Administration of Quality Supervision Inspection and Quarantine and decided to recall 197 vehicles according to the requirements of the Administrative Regulations on the Recall of Defective Automobile Products
	SAIC Motor Corporation Limited	2021	SAIC General Motors Co., Ltd. Recalls Imported Cadillac Carrera
**others**	Chow Tai Seng Jewellery Co., Ltd.	2019	The gold products sold by Chow Tai Sang counter in Chongde Mall have weight deviation
	Midea Group Co., Ltd.	2020	The article "Midea gas stove suddenly explodes" was posted on the consumer’s microblog

## Method

This article applied event study method to estimate the impact of different strategies on the firm’s value at the onset of product-harm crisis, including at disclosure stage and initial response stage, by calculating the abnormal stock returns (CAR). At disclosure stage, we set the disclosure date as the event day t = 0, and calculated the CAR of the crisis firm to assess the different influence of disclosure strategies (proactive strategy and passive strategy) at the onset of product-harm crisis on the firm’s value. At initial response stage, we firstly use a 2(deny vs accept) *2(recall vs no recall) factorial model to measure the firms’ crisis response strategy, aiming to judge the firms’ attitude towards a product-harm crisis. Then, we designated the first response date as the event day (t = 0) and calculated CAR of the crisis-affected. This assessment enabled us to evaluate the impact of different response strategies in mitigating the negative consequences caused by a product-harm crisis.

## Result

### Financial performance of a product-harm crisis disclosure

This study set estimation period t = -200 to t = -20 [11, 25], relative to the event day t = 0 (we called Day 0). Day 0 is the date when a product-harm crisis event was disclosed. As the Table 2 shows, when a product-harm crisis was disclosed (Day 0), the result of average abnormal return was -0.39% and significant statistically. It suggested the product-harm crisis brought negative impact on the firm’s value with the stock price declining. What is more, in the following days (Day 1 and Day 2), the negative impact continue to exist significantly. On the days before the crisis exposed (Day -5 to Day -1), the average abnormal returns varied without statistical significance, so the corresponding CAR values are not counted. In summary, it indicated the negative AAR on Day 0 should be the attribution of product-harm crisis. H_1_ was supported.

**Table 2:** Event study result on disclosure date.

Day	average abnormal return (AAR)	cumulative abnormal return (CAR)
-5	0.01%(0.07)	
-4	0.09%(0.44)	
-3	0.07%(0.34)	
-2	0.39%(1.90*)	
-1	-0.02%(-0.12)	
0	-0.39%(-1.86*)	-0.39%(-1.86*)
1	-0.45%(-2.18**)	-0.84%(-2.81***)
2	-0.52%z(-2.52**)	-1.36%(-3.72***)

*p < .10

**p < .05

***p < .01.

### Proactive strategy and passive strategy at disclosure stage

As the product-harm crises bring negative impact on the firm’s value on the stock market at the onset, managers need to make appropriate strategies to diminish the adverse influence. In line with Chen et al. [18], we classify the disclosure strategy into proactive strategy and passive strategy. Proactive strategy indicated the firm disclosed its product-harm crisis new before the media, or other subjects. Passive strategy implied the product-harm crisis news was released by the media, consumers’ complaint, or regulatory authorities, instead of by the firm itself.

We set estimation period t = -200 to t = -20, relative to the event day t = 0 (we called Day 0). Day 0 is the date when the crisis event was disclosed. We concentrated on the cumulative abnormal returns (CAR), from the event window t = 0, to t = +2, capturing delay if investors use only follow-up information. CAR (0,+2) is defined as the sum of the differences between actual and expected stock returns on days t = 0, +1, +2.

Table 3 shows CAR of proactive strategy and passive strategy at disclosure stage, CAR (0,2) of passive strategy is -1.09%, with 141 observations, and CAR (0,2) of proactive strategy is -1.74%, with 28 observations. It demonstrated that most product-harm crisis managers preferred to take passive strategy at disclosure stage and proactive strategy was associated with a more negative cumulative abnormal return than passive strategy. H_2_ is supported.

**Table 3:** CAR of proactive strategy and passive strategy at disclosure stage.

Disclosure strategy	CAR (0,2)	Firm types	CAR (0,2)
**Passive strategy (**141 observations**)**	-1.09%***	Without product-harm crisis history (52 observations)	-0.89%***
		With product-harm crisis history (89 observations)	-1.21%***
**Proactive strategy (**28 observations**)**	-1.74%*		

*p < .10

**p < .05

***p < .01.

### Matched response strategies at initial response stage

Learning from views of [8], we design a 2 (deny vs accept) *2 (recall vs no recall) factorial model to distinguish firms’ attitude towards the product-harm crisis. Response strategy with both accept and product recall is acknowledged as overconforming strategy, due to taking too much responsibility. Response strategy with both deny and no product recall is recognized as underconforming strategy, for taking inadequate responsibility. Response strategy of accept+no product recall and deny+product recall are recognized as conforming strategy. Table 4 lists the firms’ response strategy matching and cumulative abnormal stock returns. The results of CAR(-1,1) are significant (p<0.01). It shows that conforming strategies receives more positive CAR than underconforming strategies and overconforming strategies. Conforming strategies including conforming 1 and conforming 2, receives CAR of -0.83% and -1.74% respectively, while underconforming strategies receive CAR of -2.23%, overconforming strategies receive CAR of -2.91%. H_3_ is supported.

**Table 4:** Response strategy match and cumulative abnormal stock returns.

Response strategies	CAR (-1,1)		
	All observations	Firms with product-harm crisis history	Firms without product-harm crisis history
**Accept+recall (Overconforming)**	-2.91%***	-2.28%***	-4.11%***
**Accept+no recall (Conforming 1)**	-0.83%***	-2.97%***	-1.39%***
**Deny+recall (Conforming2)**	-1.74%***	-2.13%***	-0.83%***
**Deny+no recall (Underconforming)**	-2.23%***	-2.16%***	-1.56%***

*p < .10

**p < .05

***p < .01.

### Robustness test

Firstly, according to Fan [23], we apply Market-Adjusted Model in Event Study Method to re-estiamate the CAR at disclosure stage and initial response stage, with the estimation of window of [–200,–20]. Table 5 show the results, and we find that the CARs are still significant.

**Table 5:** Estimation of market-adjusted model in event study method.

Stages of Response	Response Strategies	CAR	No. Of Observations	P-value
**Disclosure Stage**	Passive Strategy	-1.50%	141	0.00
**Initial response stage**	Four Types of Response Strategies	-2.18%	106	0.00

Secondly, we adjust the estimation window to (-110, -10) to determine whether the result of stock market reaction to the strategies taken at disclosure stage and initial response stage are still significant. Firstly, at disclosure stage, we get the significant result of CAR(0,2) = -1.67%, p = 0.06 for the proactive strategy and CAR(0,2) = -1.19%, p = 0.00 for the passive strategy. Then, we also get the significant result of CAR(-1,1) = -0.025, p = 0.00, at initial response stage. All results are still statistically significant and illustrates the robustness of our findings in this article.

Finally, referring to Wang et al. [48], we conducts time-shifted placebo test by adjusting the exposure date and response date of each product-harm crise (adding 10 days to the original exposure date and response date respectively), and re-estimate the corresponding CAR. As shown in Table 6, the results are all insignificant at disclosure stage and initial response stage (with CAR (0, 2) = -1.20%, p = 0.2056 for the proactive strategy; CAR = -1.60%, p = 0.7181 for the passive strategy; CAR(-1,1) = 0.03%, p = 0.9595 for four types of response strategies). The above results again prove the robustness of our findings.

**Table 6:** Time-shifted placebo tests for exposure date and response date.

Time Shift	Stages of Response	Response Strategies	CAR	No. Of Observations	P-value
Adding 20 days to exposure date	Disclosure Stage	Proactive Strategy	-1.20%	28	0.2056
		Passive Strategy	-1.60%	141	0.7181
Adding 20 days to response date	Initial response stage	Four Types of Response Strategies	0.03%	106	0.9595

### Additional analysis: Crisis history as an intensifier

As SCCT emphasized, crisis history was an intensified factor of responsibility attributions, which would increase the level of attributions in a crisis [8]. Due to the negative impact of crisis history on the crisis strategy effectiveness, crisis managers who strove to make matched response strategy should take more responsibility. We adopt empirical data to verify this viewpoint by comparing the different stock market performances of the same crisis response strategy applied in two types of samples with and without crisis history.

### Impact of crisis history at disclosure stage

As we found almost all product-harm crisis firms who applied proactive strategy at disclosure stage have product-harm crisis history. In this situation, we cannot distinguish firm types to determine the impact of crisis history. So we focus on samples which adopt passive strategy at disclosure stage. It is showed in Table 3, for the passive strategy at disclosure stage, the firm without product-harm crisis history (CAR is -0.89%) gains better strategy effect than the firms with crisis history (CAR is -1.21%). H_4_ is supported.

### Impact of crisis history at initial response stage

As Coomb [8] proposed that firm with product-harm crisis history before should take more responsibility than firm without product-harm crisis. We exam this viewpoint from the listed firms’ product-harm crises in China. We find that, when taking conforming strategies, firms without product-harm crisis received better market reaction than firms with product crisis before. As showed in Table 4, in the situation of conforming 1 and conforming 2, firms without product crisis history have less market value loss than firms with product crisis history (CAR of firm without product crisis history is -1.39%, -0.83% respectively, while CAR of firm with product crisis history is -2.97%, -2.13% respectively). In addition, when firms take excessive responsibility (overconforming strategy), market reaction of firms with product-harm crisis history is better than that of firms without product crisis history (CAR of firm with product crisis history is -2.28%, CAR of firm without product crisis history is -4.11%). When firms take insufficient responsibility, the result is opposite. All above indicated that, comparing with firms without product-harm crisis history, firms with product-harm crisis history will be associated with less negative cumulative abnormal return when they adopt crisis response strategy of taking too much responsibility, while they will be associated with more negative cumulative abnormal return when they adopt response strategy of taking too little responsibility. H_4_ is supported.

## Conclusion and discussion

This article investigates stock market reactions to various crisis response strategies, aiming to offer crisis managers valuable recommendations for developing effective strategies during product-harm crises. Firstly, we find that proactive strategy at disclosure stage brings a more negative impact on crisis firm’s stock market value than passive strategy in a product-harm crisis. This is consistent with some previous scholars who have argued that proactive disclosure of negative information have a more adverse impact in the short term, mainly in the form of negative abnormal returns on the stock market [20, 49]. However, some scholars proposed different viewpoint that proactive disclosure strategy in a crisis is more effective than passive disclosure strategy [16, 36, 50, 51]. They believed that proactive strategy can give real information at the first time to the stakeholders, and gain their trust, which can effectively minimize the reputation loss. The reason for the divergence of views is probably the different lengths of time, with stock market reactions considering short-term effect, while reputation and trust considering long-term effect. Secondly, our findings suggest that response strategies of accept+no recall and deny+recall are considered conforming and effective at the onset of a product-harm crisis. In contrast, accept+recall strategies imply taking on too much responsibility and deny+ no recall strategies imply taking on too little responsibility. This is in line with the crisis matching strategy proposed by the SCCT theory, whereby a firm’s assumption of responsibility in a crisis should be matched with its crisis attribution. Finally, consistent with previous scholars’ proposals, stakeholders attribute greater responsibility for a crisis to the firm itself if it has a history of past crises. Therefore, firms with a crisis history should assume more responsibilities. Our study demonstrates that more proactive and responsible strategies, such as proactive strategy and accept+ recall strategies, brought less negative stock market reaction for firms with crisis history.

Our research indicates that considering the stock market reaction, it is not a wise choice for crisis managers to choose a proactive strategy at disclosure stage; instead, a passive strategy is relatively appropriate. Proactive strategies are more likely to attract investors’ attention and scrutiny [18], and negative information of crisis will be reflected in the stock market [30]. Firstly, investors will view proactive strategy as that the firm cannot control the product-harm crisis but only to reveal it to the public actively [18]. Secondly, investors assumed firm’s measure to deal with the product-harm crisis will cause a large economic loss and damage their interests. Moreover, the product’s severe consequence will make investors suspect of the firm’s operating ability and weaken investors’ confidence [52]. The above implies that passive exposure strategies are more appropriate from an investor’s perspective.

Our research also shows that crisis managers can reduce the negative stock market reaction at the onset of a product-harm crisis by adopting conforming response strategies, such as accept+no recall and deny+recall. Both strategies imply that the responsibility assumed are conforming and matched with crisis attributions [8, 18].

In addition, our research also implies that firms with crisis history should choose a response strategy that assumes more responsibility in handling a new product-harm crisis, where a seemingly overconforming strategy such as (Accept+recall) seems more appropriate. Since crisis history is a reinforcing factor in crisis attribution in crises [8], the public tends to attribute more responsibility to the firm who have a product-harm crisis again. Therefore, crisis managers should opt for a response strategy that assumes more responsibility at this juncture, with the accept+recall response strategy being the more appropriate choice.

### Limitations and future studies

The first limitation is that we only study the short-term effects of firms’ crisis coping strategies from the perspective of stock market reactions, focusing on appropriate crisis strategies at the onset of product-harm crisis. However, as scholars have proposed, the short-term effects of crisis response strategies (e.g., product recall strategies) on firms can be different from the long-term effects [32]. Future research can be based on a developmental perspective to dynamically analyze the long-term effects of crisis response strategies on firms during the development of a product-harm crisis.

The second limitation is that this study did not take into account the different industry characteristics in which the crisis firms are located, due to the relatively small sample size. Future research will consider industry heterogeneity and analyze the different effect of crisis response strategies in product-harm crises in different industries.

The third limitation is that we only considered response strategies in product-harm crisis situation. Future research will expand the crisis scenarios to include safety accident crises, environmental pollution crises.
